# Extending the range of additivity in using inclusive fitness

**DOI:** 10.1002/ece3.6935

**Published:** 2021-02-07

**Authors:** Samuel R. Levin, Alan Grafen

**Affiliations:** ^1^ Department of Zoology Oxford University Oxford UK; ^2^ St John's College Oxford University Oxford UK

**Keywords:** fitness, kin selection, natural selection, population genetics

## Abstract

Inclusive fitness is a concept widely utilized by social biologists as the quantity organisms appear designed to maximize. However, inclusive fitness theory has long been criticized on the (uncontested) grounds that other quantities, such as offspring number, predict gene frequency changes accurately in a wider range of mathematical models. Here, we articulate a set of modeling assumptions that extend the range of scenarios in which inclusive fitness can be applied. We reanalyze recent formal analyses that searched for, but did not find, inclusive fitness maximization. We show (a) that previous models have not used Hamilton's definition of inclusive fitness, (b) a reinterpretation of Hamilton's definition that makes it usable in this context, and (c) that under the assumption of probabilistic mixing of phenotypes, inclusive fitness is indeed maximized in these models. We also show how to understand mathematically, and at an individual level, the definition of inclusive fitness, in an explicit population genetic model in which exact additivity is not assumed. We hope that in articulating these modeling assumptions and providing formal support for inclusive fitness maximization, we help bridge the gap between empiricists and theoreticians, which in some ways has been widening, demonstrating to mathematicians why biologists are content to use inclusive fitness, and offering one way to utilize inclusive fitness in general models of social behavior.

## Graphical abstract

Inclusive fitness has long been criticized on the grounds that other quantities, such as offspring number, predict gene frequency change in a wider range of mathematical models. We show how the range of applicability of inclusive fitness can be extended, offering one way to utilize inclusive fitness in a wider range of models, and further justification for empiricist's use of inclusive fitness.

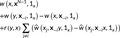

## 1 INTRODUCTION

Inclusive fitness is an individual‐level quantity identified by Hamilton (1964), which he showed, under some assumptions, to increase due to the action of natural selection. Hamilton genetical pointed out that adult offspring number is affected not just by the actions of an individual but by those of the individuals it interacts with. He observed that measuring those effects involves averaging over possible distributions of genotypes, which in turn involves knowing gene frequencies in the population, neither of which are simple or readily available calculations (Hamilton, 1964). Accordingly, he turned to an alternative metric, “inclusive fitness,” which involves taking the perspective of the focal individual and its effects on others (as opposed to others’ effects on it).

Hamilton (1964) provided a verbal definition for inclusive fitness as follows: the sum of an individual's adult number of offspring after it has been “stripped of all components which can be considered as due to the individual's social environment,” and a weighted sum of the “quantities of harm and benefit which the individual himself causes” to the offspring numbers of others. The weightings are degrees of relatedness. Relatedness is a measure of genetic similarity between two individuals (r = 1 for identical twins, r = 0 for random population member, including possibility of self in finite populations). The exact definitions of the fitness effects and of relatedness differ in different formal treatments. For nearly 40 years, at least within behavioral and evolutionary ecology, most field and laboratory workers have treated inclusive fitness as the quantity that organisms appear designed to maximize, and tailored their studies and experiments accordingly (summarized in, e.g., Davies et al., 2012; Westneat & Fox, 2010). However, Hamilton's verbal definition lacks mathematical precision, and in Section 4.2 below we provide such precision for a particular model. This is important for reconciling relatively informal inclusive fitness arguments with full population genetic models.

Further, since at least 1978 (Cavalli‐Sforza & Feldman, 1978), the concept of inclusive fitness has been controversial, criticized most notably for assuming additivity of fitness effects. The type of additivity we discuss here refers to how the effects of different social actions combine to affect one individual's offspring number. The well‐known challenge for inclusive fitness is that under nonadditivity, changes in gene frequency are no longer wholly attributable to a focal genotype. Since at least 1979 authors have pointed out that, in such scenarios, mean offspring number does a better job at predicting gene frequency change (Grafen, 1979). Unfortunately for biologists, mean offspring number is not a useful maximand in practice, either in terms of empirical applicability or explanatory power (discussed in detail by Levin & Grafen, 2019). Therefore, the problem of nonadditivity remains a relevant challenge for empirical biology.

A potential solution to the problem of nonadditivity is weak selection. Weak selection can arise either because the contributions to fitness of a mutation are relatively small (“w‐weak selection”) or because the mutant is not far from the wild‐type in phenotype space (on average, “δ‐weak selection”). There is a wide consensus that under weak selection organisms at equilibrium act as if maximizing their inclusive fitness (Gardner et al., 2011; Grafen, 2006; Lehmann et al., 2015, 2016; Lehmann & Rousset, 2014; Okasha & Martens, 2016b; Taylor, 2017). While this goes some way toward satisfying biologists hoping to use inclusive fitness, two significant challenges remain. First, the difficulty of capturing Hamilton's inclusive fitness in models has forced many mathematical biologists to use replacements for the correct inclusive fitness, such as “simple‐weighted sum” inclusive fitness, or neighbor‐modulated fitness, in their tests for maximization (Lehmann et al., 2015; Okasha & Martens, 2016b). This makes the results for inclusive fitness maximization, positive or negative, difficult to interpret biologically. Second, it is not immediately clear how widely we expect weak selection to hold. If the conditions needed for inclusive fitness maximization are rare in practice, this would be unfortunate news for empiricists, as no practically useful alternative maximand has been offered (Levin & Grafen, 2019).

Our aim here, then, is to resolve both problems, providing formal support for extending the range of inclusive fitness's applicability. We do this through two steps. First, we invoke an assumption that we expect to be reasonable and usually close to holding across a wide range of biological scenarios, and which recovers a form of weak selection. This is the assumption that the strategy set (set of possible phenotypes) contains all probabilistic mixtures of all pairs of strategies. Second, we illustrate a new method for capturing Hamilton's (1964) verbal definition of inclusive fitness in a mathematical model testing for fitness maximization, which is to replace Hamilton's comparison with the nonsocial situation with a comparison with the resident phenotype, thus taking an ESS‐like approach. Using these two steps, we show that inclusive fitness is indeed maximized under probabilistic mixing in two particular models. This provides formal support for biologically meaningful extension of the range of applicability of inclusive fitness, and a mathematical method for utilizing Hamilton's inclusive fitness in maximization modeling.

We proceed as follows. First, we illustrate the two steps in greater detail, providing verbal arguments for the importance of probabilistic mixing and correctly capturing inclusive fitness. Second, we turn to two recent models by Okasha and Martens (2016b) and Lehmann et al. (2015) does which developed sophisticated techniques for studying inclusive fitness maximization. These two models are of particular interest because they study fitness maximization at the individual level in an encouraging way, and yet do not find inclusive fitness maximization where biologists would hope they might. We reanalyze these models, showing that our suggested new assumption of probabilistic mixtures and suggested new expression for inclusive fitness recover inclusive fitness maximization in both settings. Finally, we discuss the relevance of these findings to recent work on fitness maximization more generally, further implications of our analysis for calculating inclusive fitness, and how empirical biologists might utilize the results.

## 2 PROBABILISTIC MIXING AND INCLUSIVE FITNESS

Here, we verbally articulate two steps which recover inclusive fitness maximization in a wide range of scenarios. These points are illustrated mathematically in the subsequent sections, in which we instantiate the points in specific models.

### 2.1 Probabilistic mixing and weak selection

Our first point is that when the strategy set (set of possible phenotypes) contains all probabilistic mixtures of all pairs of strategies, weak selection arises near an equilibrium, and therefore, inclusive fitness should be maximized. To understand this point, it is useful to make a distinction between *phenotypic* and *genetic* additivity, in the following senses. Phenotypic additivity is determined by the game matrix that would be constructed by biologists studying interactions, which they interpret as a game, who can observe the actions performed in the game and the payoffs from the game but not the genotypes. Genetic additivity among a set of genotypes is determined by whether the fitnesses of individuals can be written as an additive function of the genotypes of the interactants. Weak selection arises when an analysis restricts itself to a genetically additive subset of genotypes.

We argue that under probabilistic mixing, phenotypic nonadditivity is compatible with genetic additivity. This arises because, while deviant behaviors can have large effects, they are expressed rarely. The verbal argument has been made elsewhere (Queller, 1996; Levin and Grafen, 2019 and Grafen, 1979, final paragraph on p. 906), but we repeat it here for clarity. For example, consider the case where strategies are not discrete but continuous, where a player can choose to cooperate on a fraction πof occasions. Now, a variant strategy plays Cooperate on a fraction π+δ of occasions, where δ≪1. In other words, it plays Cooperate instead of Defect on one occasion out of many, and the probability that it is the same occasion its related partner also plays Cooperate is very low (Grafen, 1979). Thus, under biologically relevant scenarios, phenotypic nonadditivity is compatible with genetic additivity (although a formal treatment has thus far been lacking).

### 2.2 Measuring inclusive fitness

We also make two significant points about the definition of inclusive fitness. One is simply to emphasize the distinction between inclusive fitness and neighbor‐modulated fitness. Inclusive fitness requires a careful isolation of the effects of actor, whereas neighbor‐modulated fitness is simply a measure of mean offspring number. These differences are important in practice for biologists and cannot simply be used interchangeably (Levin & Grafen, 2019).

However, they are often treated as synonymous in the technical literature, in part because neighbor‐modulated fitness is easier to use in mathematical models testing for maximization. For example, Lehmann et al. (2015) and Lehmann et al. (2016) discuss inclusive fitness maximization in different formal contexts, when the quantity they work with is neighbor‐modulated fitness. In their setting, while the mutant's frequency remains at zero, the average of one equals the average of the other. However, in general, they are not equal, and Hamilton (1964)'s diluting factor shows there is a difference. Their results then, while valuable for mathematical biologists, are difficult to interpret for biologists interested in utilizing inclusive fitness.

The second point is to make a helpful correction to Hamilton (1964)'s verbal definition of inclusive fitness. Costs and benefits are defined as differences from what would be expected in the nonsocial case, and this has proved a difficulty in interpretation. We suggest that the verbal definition is more useful, and more in keeping with Hamilton's mathematical definition, if the comparison is instead made with the incumbent behavior (in an ESS‐like analysis that tests for rare mutants against an incumbent) or, more generally, is made with the average behavior (which will therefore vary with gene frequencies). This suggestion makes the verbal definition easier to apply and, as we shall see, does support inclusive fitness maximization.

We now turn to two recent models (Lehmann et al., 2015; Okasha & Martens, 2016b), in an attempt to formalize these points and provide support for the use of inclusive fitness as a biological maximand. These two papers continue the encouraging trend toward an explicit mathematical treatment of inclusive fitness maximization. Although they fail to find it, and instead show that mean offspring number (in the guise of neighbor‐modulated fitness, the calculation Hamilton termed “unwieldy”) is maximized, we are able to utilize their mathematical advances to bolster the use of inclusive fitness by biologists.

## 3 A SIMPLE TWO‐PLAYER GAME

Okasha and Martens (2016b) analyze a version of the Hawk‐Dove game played between relatives (they focus on the simpler cooperation game, but we keep the discussion general here as the conclusions hold for both). Their goal was to look with mathematical precision at the question of whether inclusive fitness appears to be maximized by individuals at equilibrium. Our first point is that neither of the two fitness functions they define corresponds to Hamilton's inclusive fitness, and we show what the third function is below. Our second point is that, when we allow all probabilistic mixtures of Okasha and Martens’ strategies also to be strategies, this third function is indeed maximized.

### 3.1 Do they consider inclusive fitness?

Okasha and Martens’ (2016b) first utility function, which they refer to as inclusive fitness, is.

(1) $$U\left(i,j\right)=V\left(i,j\right)+rV\left(j,i\right),$$

where *r* is relatedness, and *V*(*i*,*j*) is an individual's payoff when playing strategy *i* against a partner who plays *j*. It is immediately apparent that this is not inclusive fitness, but something more akin to simple‐weighted sum fitness (Grafen, 1982). It measures the actor's whole payoff plus *r* times its partner's whole payoff and, therefore, does not partition offspring number by causation. They find that this utility function is not maximized, which is not surprising, as it is not inclusive fitness.

Their second utility function, which they call “Grafen, 1979,” is expressed as follows:

(2) $$U\left(i,j\right)=rV\left(i,i\right)+\left(1‐r\right)V\left(i,j\right).$$

This payoff function, identified by Grafen (1979), is simply mean number of offspring, and, as expected, Okasha and Martens (2016b) find that that the strategy with the highest value increases in frequency, and establish links between evolutionary dynamics and as‐if maximization. Clearly, neither of these utility functions is inclusive fitness as defined by Hamilton (1964).

### 3.2 What is the correct expression for inclusive fitness?

In order to ask whether inclusive fitness is maximized, we must write a third utility function, which sums the effect on personal payoff of expressing the strategy and the relatedness weighted difference to partner's payoff as a result of actor expressing the strategy, according to Hamilton's, 1964 definition. To do this, we write *k* as a default, “nonsocial” strategy and, therefore, can express inclusive fitness of an individual playing *i* against a partner playing *j* in a population (the “nonsocial strategy”) playing *k*, as the sum of the nonsocial payoff against itself, the deviation from the play of *i* rather than *k* against *j*, and relatedness times the effect on the partner of the play of *i* rather than *k*:

(3) $$V\left(k,k\right)+\left(V\left(i,j\right)‐V\left(k,j\right)\right)+r\left(V\left(j,i\right)‐V\left(j,k\right)\right).$$

This formula would be useful if required for connecting to gene frequencies using the Price equation at any frequency (Grafen, 2006), but with the probabilistic mixing assumption for invasion of an incumbent, we regard the partner as also playing the incumbent strategy, so *j* = *k* and we obtain

(4) $$U\left(i,j\right)=V\left(i,j\right)+r\left(V\left(j,i\right)‐V\left(j,j\right)\right).$$

### 3.3 Is inclusive fitness maximized under probabilistic mixing?

The problem of nonadditivity remains. Consider the simple two‐player cooperation game with discrete strategies, analyzed above, where each player can choose to play either Cooperate or Defect. Relatedness, *r*, is the measure of genetic similarity between players discussed above. In a simple two‐player game like this, *r* also measures assortation between strategies. If we imagine a mutant in the population that played Cooperate instead of Defect, increasing *r* increases the likelihood that its partner's strategy will also be Cooperate, and inclusive fitness fails to take this alteration in the partner's behavior into account. When fitness effects depend on the partner's genotype, as in the case of nonadditivity, this oversight matters.

However, when we assume probabilistic mixing, for reasons outlined above and elsewhere (Grafen, 1979; Levin & Grafen, 2019; Queller, 1996), we can recover inclusive fitness maximization. Grafen (1979, p.907) has already shown that, when we allow for probabilistic mixing of strategies, inclusive fitness correctly predicts the direction of gene frequency change in the simple game above, and this resolves the problem identified by Okasha and Martens (2016b). In 12, we provide a proof for this simple cooperation game, recovering the links between as‐if inclusive fitness maximization and gene frequency change.

In summary, Okasha and Martens’ (2016b) “inclusive fitness” function is not inclusive fitness. The natural expression for inclusive fitness arising from Hamilton's (1964) definition and our suggested amendments is our Equation (4). Under probabilistic mixing, this correct inclusive fitness is indeed maximized at equilibrium by each individual, regarding the incumbent strategy as fixed.

## 4 A GENERAL INFINITE ISLAND MODEL

The game analyzed above is a simple two‐player game. In some ways, this is very general, because it allows us to make few assumptions about life cycle or population structure (namely that the chance of meeting an identical strategy depends only on *r* and *p*, which are both independent). However, it is a restricted sort of interaction, in which *r* is a parameter rather than an endogenous feature of the model. We now turn to a recent rigorous population genetic analysis, which is in some ways more general, and which makes similar claims to Okasha and Martens (2016).

Lehmann et al. (2015) consider an infinite island model of haploid individuals on patches of a fixed size. An individual's fitness is a function of its own strategy and the profile of strategies to be found among its neighbors, where we will need to refer to their general space results, but for the moment focus on the case where the strategy set is confined to real numbers. There is assumed to be no class structure, and there is permutation invariance of fitness as a function of the nonself elements of the profile of neighbor strategies (which rules out associating with relatives more than associating with random members of the group). Individuals are asexual, and offspring migrate with some positive probability. Generations may be discrete or overlapping, but adults do not migrate. Otherwise, no assumptions are made about fecundity, survival, or competition. This allows for any type of games to be played on the patches and any type of strategies to be employed. Thus, despite the highly specific population structure, the model is otherwise quite general. Accordingly, any conclusions drawn from the model about the maximization of inclusive fitness are of interest.

The approach is then as follows. Consider a mutant individual and the conditions that must hold for this mutant strategy to invade the population. In an infinite island model, any mutant will either ultimately go extinct or go to fixation in the population. Thus, we can identify the uninvadability condition for a strategy. The question then becomes whether we can construct an individually defined payoff that depends on the individual's own strategy and also on that of its fellow group members, and identify the payoff with a form of biological fitness, such that strategies that maximize the expected value of this payoff against a population almost all playing its own strategy, with group membership determined by the population structure, are also the strategies that satisfy the uninvadibility criterion.

### 4.1 Do they consider inclusive fitness?

Lehmann et al. (2015) define three such candidate utility functions, but our first point will be that none of them corresponds to Hamilton's verbal definition of inclusive fitness with our interpretive principle, a fourth function that we exhibit below. Our second point is that, once we allow probabilistic mixtures of Lehmann et al.'s strategies also to be strategies, this fourth function is indeed maximized at equilibrium. Some of Lehmann et al.'s arguments apply to general strategy sets, and these already include probabilistic mixtures. When we come to the parts that focus on simple real number strategies, we will need to extend the domain of fitness and other functions accordingly.

First, Lehmann et al. (2015) identify the utility function *u_A_*, which they refer to as inclusive fitness:

(5) $$u_{A}\left(x_{i},x_{‐i},1_{x}\right)=w\left(x_{i},x_{‐i},1_{x}\right)+r\left(\bar{x},\bar{x}\right)\sum_{j\neq i}w\left(x_{j},x_{‐j},1_{x}\right).$$

$w\left(x_{i},x_{‐i},1_{x}\right)$ is the offspring number of an focal individual, *i*, expressing strategy *x_i_* in a patch where the strategies of the individuals other than *i* are represented as **x**_−_ *_i_*, where, recalling that the mutant is at zero frequency, the distribution of the whole population (1*_x_*) is assumed to be monomorphic for *x* and $r\left(\bar{x},\bar{x}\right)$ is relatedness (with $\bar{x}$ being the average strategy in the population). It is immediately apparent then that *u_A_* is not inclusive fitness, but instead a version of “simple‐weighted sum” fitness (Grafen, 1982). It measures an individual's personal offspring number plus a weighted sum of the offspring of all its social interactants and therefore fails to isolate the actor's effects, as Hamilton (1964) intended.

Lehmann et al. (2015) then turn to a second utility function, *u_B_*, which they refer to as “average personal fitness,”

(6) $$u_{B}\left(x_{i},x_{‐i},1_{x}\right)=\sum_{k=1}^{N}\sum_{{\tilde{x}}_{‐i}\in P_{k}\left(x_{‐i}\right)}w\left(x_{i},{\tilde{x}}_{‐i},1_{x}\right)q_{k}\left(\bar{x},\bar{x}\right),$$

where *P_k_* is the subset of hypothetical neighbor strategy profiles such that *k* − 1 neighbors have a strategy identical to the focal individual, and $q_{k}$ is the probability of that profile (Lehmann et al., 2015). *u_B_* is a version of neighbor‐modulated fitness (i.e., simply mean offspring number), as it counts an individual's offspring number incorporating the effects of its social partners. Note, of course, that Okasha and Martens’ “Grafen, 1979” payoff (our Equation 2) is the simple two‐player game version of Lehmann et al.'s *u_B_* (our Equation 6), as both are mean offspring number. Lehmann et al. consider a third function, *u_C_*, which we do not reproduce here as it is simply neighbor‐modulated fitness under a special assumption about the link between offspring number and material payoffs.

Lehmann et al. find a much closer fit between the uninvadability conditions from the dynamic model and the first and second order conditions for “as‐if” maximization by individuals for *u_B_* than *u_A_* (Lehmann et al., 2015). This is not surprising, as we expect this to hold for mean offspring number, and it parallels Okasha and Martens’ finding about Grafen, 1979. However, none of these functions is inclusive fitness as Hamilton (1964) outlined, and therefore, their analysis cannot satisfactorily interrogate inclusive fitness maximization.

### 4.2 What is the correct expression for individual‐level inclusive fitness?

Instead, we require a fourth function, which we will call *u_IF_*. In line with Hamilton (1964), to obtain *u_IF,_* we must sum three components: baseline asocial fitness, the difference to personal fitness as a result of the strategy, and relatedness weighted difference to social partners’ fitnesses as a result of the strategy. We define the inclusive fitness of a player with the focal strategy, in a group with an arbitrary distribution of other strategies, but in a population in which almost all individuals play an incumbent strategy *x*. This follows the individual‐level philosophy as outlined by Lehmann et al. (2016). We will go on to convert that expression to investigate the invader‐incumbent case, following Lehmann et al. (2015). Recalling our principle, from the previous example, of adopting the incumbent as the nonsocial strategy for inclusive fitness purposes, inclusive fitness is made up of the following parts:

- Baseline asocial fitness in the population as a whole – the average for an x‐player, so

$$w\left(x,x^{N‐1},1_{x}\right),$$

where **x** *^N^* ^−1^ indicates that all other group members play *x*,

- The difference to own personal fitness as a result of being a *y*‐strategist rather than an *x*‐strategist, in which others play an arbitrary (*N*−1)‐tuple of strategies **x**_−_ *_i_*:

$$+w\left(y,x_{‐i},1_{x}\right)‐w\left(x,x_{‐i},1_{x}\right).$$

- The difference to others’ personal fitnesses as a result of the focal individual being a y‐strategist rather than an x‐strategist, weighted by relatedness:

$$+r\left(y,x\right)\sum_{j\neq i}\left(\hat{w}\left(x_{j},x_{‐i}y,1_{x}\right)‐\hat{w}\left(x_{j},x_{‐i}x,1_{x}\right)\right),$$

where $\hat{w}$ differs from w in that the second argument of $\hat{w}$ describes the strategies of the whole group, and not of the group apart from *i*. Formally, $w\left(x,z,1_{x}\right)=\hat{w}\left(x,zx,1_{x}\right)$, and we regard $\hat{w}$ as being undefined if the first argument is not also an element in the group strategies. $r\left(y,x\right)$ is relatedness from the perspective of a *y* player in a population of resident *x* players. *x_j_* for j≠i are the elements of **x**_−_ *_i_*.

Putting all this together, we can write the inclusive fitness of an individual playing y, in a group $x_{‐i}$, with population incumbent *x* as follows:

(7) $$\begin{array}{c} w\left(x,x^{N‐1},1_{x}\right)\\ +w\left(y,x_{‐i},1_{x}\right)‐w\left(x,x_{‐i},1_{x}\right)\\ +r\left(y,x\right)\sum_{j\neq i}\left(\hat{w}\left(x_{j},x_{‐i}y,1_{x}\right)‐\hat{w}\left(x_{j},x_{‐i}x,1_{x}\right)\right). \end{array}$$

If using this expression to understand gene frequencies in general, we would average this expression over the distribution of **x**_−_ *_i_* that the population structure implies. If instead we are testing for invasion of a population playing xby a rare mutant playingy, all individuals would be playing *x* ory, and this would allow us to write $x_{‐i}=y^{\left(k‐1\right)}x^{\left(N‐k\right)}$ for a group with kmutants altogether, and average over the different values of *k* with their probabilities $q_{k}\left(y,x\right)$ in Lehmann et al.'s notation. Going further, under the probabilistic mixing assumption, as already discussed in relation to the Okasha and Martens model, we would evaluate inclusive fitness substituting **x** ^(^ *^N^* ^−1)^ for **x**_−_ *_i_*, that is, we would assume that the neighbors were all playing the incumbent strategy whether they were genetically mutant or genetically incumbent. This simplifies Equation (7) and allows us to define inclusive fitness under probabilistic mixing for invasion‐incumbent purposes as

(8) $$\begin{array}{cc} u_{\mathrm{IF}}\left(y,x\right)= & w\left(y,x^{\left(N‐1\right)},1_{x}\right)\\ & +\left(N‐1\right)r\left(y,x\right)\left[w\left(x,x^{\left(N‐2\right)}y,1_{x}\right)‐w\left(x,x^{\left(N‐1\right)},1_{x}\right)\right]. \end{array}$$

This simple form is a way of applying additive ideas to phenotypically nonadditive situations and recovers much of the simplicity of the additive case.

### 4.3 Is inclusive fitness maximized under probabilistic mixing?

In Section 2.3, we offered a verbal argument for the biological importance of probabilistic mixing. Here, we formalize this argument by extending Lehmann et al.'s (2015) model to allow for probablistic mixing of strategies and analyze the first and second order conditions for evolutionary uninvadability and maximization of inclusive fitness ($u_{\mathrm{IF}}$). We do this by considering a mutant strategy $\tilde{y}$, in which a mutant displays the deviant behavior with some small probability, ε, and otherwise, with probability $\left(1‐\varepsilon \right)$, behaves like a resident (x), which we can write in a natural notation as $\tilde{y}=\left(1‐\varepsilon \right)x+\varepsilon y$

Formally, our probabilistic mixing assumption is that if *y* and *z* are elements of our strategy set *X*, then so is every probabilistic mixture of *y* and *z*. Thus, let $\phi \left(y,z,e\right)$ be the strategy that plays *z* with chance 1‐ε and *y* with chance ε. So far, we have been using results of Lehmann et al.'s that apply to general strategy sets. When we move to look at first and second order conditions, we follow them in moving to one‐dimensional (real) strategies, except that we need to extend the domain of some functions to include probabilistic mixtures.

Because *w* is an expected number of offspring anyway, it is reasonable to extend *w* by writing

(9) $$w\left(\phi \left(y,z,\varepsilon \right),x_{‐i},1_{x}\right)=\left(1‐\varepsilon \right)w\left(z,x_{‐i},1_{x}\right)+\varepsilon w\left(y,x_{‐i},1_{x}\right),$$

and a similar expansion applies to each of the other probabilistically mixed arguments that may appear in $x_{‐i}$, allowing us to unpack $w\left(y,x_{‐i},1_{x}\right)$ into a convex combination of the values of *w* defined for scalar strategies.

We proceed to ask whether evolutionary uninvadability = utility maximization, by checking whether the first and second order conditions for uninvadability and utility maximization are the same. Following Lehmann et al. (2015, equation (3), which applies to arbitrary strategy sets), we write the lineage fitness of the mutant as:

(10) $$W(\tilde{y},x)=\sum_{k=1}^{N}\left(\begin{array}{c} N‐1\\ k‐1 \end{array}\right)q_{k}(\tilde{y},x)w\left(\tilde{y},{\tilde{y}}^{\left(k‐1\right)}x^{\left(N‐k\right)},1_{x}\right),$$

where W is the lineage fitness of the mutant, w is the personal fitness of the mutant expressing $\tilde{y}$ in a patch with k‐1 other mutants and N‐k residents displaying x, in a population otherwise monomorphic for x. $q_{k}$ is the probability that the neighbor profile of the focal mutant will consist of k‐1 other mutants. For x to be uninvadable, it must be that $x\in \arg\max_{y}W\left(y,x\right)$, that is, x must be the best invader against itself and so must achieve a local maximum of $W\left(y,x\right)$

In the appendix, we find the first order condition for uninvadability under our probabilistic mixing condition (based on the first partial derivative of *W*) to equal the first order condition for utility maximization (based on the first partial derivative of $u_{\mathrm{IF}}$), and the same for the second order conditions. Therefore as a result of gene frequency dynamics, at equilibrium, organisms appear as if trying to maximize inclusive fitness. Due to the wide latitude afforded by the approach, this result holds some generality for inclusive fitness maximization.

In summary, Lehmann et al. (2015) does did not analyze inclusive fitness as defined by Hamilton (1964). We derive the natural expression above in Equation (8). We show in the appendix that under probabilistic mixing, the correct inclusive fitness is indeed maximized.

We expect our result to hold for other recent analyses which have identified mean offspring number as a successful maximand (e.g., Allen & Nowak, 2015), if we adopt our newly articulated modeling approach of regarding the incumbent as Hamilton's “nonsocial” case, and of allowing all probabilistic mixtures of elements in the original strategy set. An interesting future step would be to try to extend our result to more general population structures. Our articulation of these additional conditions will, we hope, help mathematicians and biologists understand each other better in future.

## 5 DISCUSSION

Inclusive fitness has formed the bedrock of a vast body of empirical literature (for an entry into that literature, see: Foster (2009); Davies et al. (2012), and for an attempt to quantify such successes Abbot et al. (2011), Tables 1 and 2). However, it has long been criticized for its assumptions, most notably additivity of fitness effects, and its failure in such scenarios to predict gene frequency change as well as mean offspring number (sometimes referred to as “neighbor‐modulated fitness”). Recent papers have apparently lent support to such claims (though this may not have been their goal) with general mathematical models (Lehmann et al., 2015; Okasha & Martens, 2016b). However, we have shown that such models fail to correctly capture inclusive fitness, and that when the correct expression is used, under the assumption of probabilistic mixtures of phenotypes inclusive fitness maximization is recovered.

### 5.1 Inclusive fitness maximization

The precise mathematical definition of inclusive fitness depends on the specific settings. However, in defining it precisely in specific cases, here we have aimed to help mathematical biologists find the precise definition in their own setting. Rousset (2004, pages 194–195) has a useful discussion of how the idea of fitness maximization can be understood mathematically, concluding that it should be understood in an ESS‐like way, considering the success of a rare mutant against an incumbent. This is in line with the approach advocated by Dawkins (1976, 1980). This implies that the fitness function must depend not only on the individual's strategy, but also on the incumbent strategy. The stable incumbent is one that is the best‐spreading mutant against itself, and the calculation of best‐spreading may rely on reproductive values in structured populations. It is useful if the definition of inclusive fitness also connects to gene frequency change at nonrare frequencies, as in Grafen (2006).

There are a number of papers that have considered inclusive fitness maximization whose work we have not addressed explicitly here, but which readers may be interested in referring to for broader considerations of biological maximization. Hamilton (1964) explicitly discusses the optimization of inclusive fitness, but this is a verbal remark drawing a parallel with the Fundamental Theorem of Natural Selection, in which Fisher (1930) also had no formal treatment of maximization. A number of papers similarly have discussions on the basis of calculations about changes in gene frequency. A recent important development is the consideration of inclusive fitness maximization at the population level by Lehmann et al. (2016), but here we have been concerned with mathematically explicit studies of inclusive fitness maximization at the level of the individual (Levin & Grafen, 2019, discuss in detail why this matters).

The first paper to do this was Grafen (2006), but his highly technical conditions, although perhaps required in a model without dynamic sufficiency, have not so far met with approval or been further developed. Second, Lehmann and Rousset (2014) fitness showed in a simple model that inclusive fitness was maximized under additivity of phenotypic effects on offspring number but not otherwise. Thus, we have focused on Lehmann et al. (2015) and Okasha and Martens (2016b), as these are the only papers we know of to explicitly analyze inclusive fitness maximization at the individual level. We note that in the case of Lehmann et al., the failure to find inclusive fitness maximization was not their main conclusion. Thus, our aim is not to say that these analyses are wrong or not useful—quite the opposite. Instead, we simply note that both papers appear to offer disappointing conclusions for users of inclusive fitness, and hitchhike on their very useful technical developments to offer further useful biological results.

In doing so, we are following a recent resolution offered by Birch (2017a,b), who argues that the critics (e.g., Allen & Nowak, 2016; Nowak et al., 2010; van Veelen et al., 2017) are right to point to technical difficulties in establishing that inclusive fitness is well‐defined or that natural selection leads to “as‐if maximization,” *in a fully general theoretical model*. However, Birch argues that, within certain assumptions, notably additivity of fitness effects, inclusive fitness is close enough to being “right” to justify its use as organizing framework for understanding social behavior. We strengthen Birch's resolution by extending the range of scenarios in which inclusive fitness can be applied. The significance of articulating modeling assumptions lies in the process by which biological ideas become transferred into mathematics. If biologists fail to explain clearly enough what they are doing, then the machinery of mathematics is capable of yielding unbiological answers that are hard for biologists to interpret or respond to. In controversies caused by failure of communication, biologists can be grateful for the work of philosophers in acting as intermediaries (Birch, 2017a; Okasha & Martens, 2016a).

We hope to have provided a way to utilize this understanding to correctly capture Hamilton's inclusive fitness in such models. The technical mathematical requirements of building rigorous population genetic models are considerable. They often require focusing on quite detailed special cases that are in themselves quite complex, or on abstract mathematical concepts representing the limits of the proof, which are quite complex, and often require adopting a very precise mathematical mode of reasoning. It is not surprising that linking back to general concepts in less technically demanding areas of biology often seems to prove difficult. In extending these models and formalizing our verbal arguments, we hope to make it easier for future modelers to make links to the general and verbally expressed conceptual theory when they build precise mathematical population genetic models.

### 5.2 Probabilistic mixing

The biological significance of the “probabilistic mixtures” assumptions is important to understand. Some of what follows is at the moment our own intuition, and obtaining mathematical proofs of precise versions would be extremely useful. First, uncontroversially, it will usually be conceivable that the assumption is true in any particular example and cannot be ruled out. Second, we conjecture that the possible deviations from the assumption will not tilt the biology in any particular direction, and thus, we can consider the equilibrium under the probabilistic mixtures assumption as a central case. The fact that this central case applies without knowledge of the genetics across such a wide range of possibilities is very important in regarding social biology as possible without detailed genetic knowledge.

The probabilistic mixing approach also provides a particular answer to a little‐discussed extra problem raised by nonlinearity. When we ask for the effect of an actor on recipients, should we ask for that effect on the basis that the recipients are (a) incumbents (b) mutants or (c) some probabilistic mixture depending on population structure and relatedness in particular? Under linearity, these all give the same answer. Eshel (2018), for example, assumes we should assume the recipients are mutants, presumably on the grounds that it is the mutant recipients that will further spread the mutant allele, but his model and our rationale for it depend on haploidy.

However, one consequence of probabilistic mixing is that inclusive fitness should be calculated on the assumption that the recipients are incumbents, and we regard this as biologically appropriate. If mutations really were unconditional, then some mixture would be preferable. But most behavior is conditional, and chance events lead individuals into expressions of different parts of a complex phenotype, so we should not expect to see a correlation of behaviors between related interactants. This chimes with our aim in the Okasha and Martens (2016) example to separate the two effects of relatedness, retaining the part that an actor “cares about” the recipient's offspring number, but ignoring the possibility that the recipient's behavior will tend to be more like the actor's than the population average. This suppression of the second effect provides a unique inclusive fitness, while the alternative is to have a complicated expression that depends on genetic details such as ploidy, penetrance, and dominance, as well as how often the genetic potential for deviant behavior is actually expressed because the appropriate environmental conditions happen to arise. This simplification may reduce the difference between the gene‐centered and individual‐centered approaches discussed by Lehmann et al. (2015) and Lehmann et al. (2016). Thus, an important question that can be asked of any inclusive fitness formulation under nonlinearity is “what is assumed about the phenotype of the recipients?” We do recognize that in this paper we have focussed only on haploidy and that further challenges are likely to arise in applying our general philosophy to diploid or mixed‐ploidy models.

Finally, when the assumption is not true, and the phenotype that would be the equilibrium under that assumption is not available as true‐breeding under the actual phenotype set, the possible outcomes are as follows. The simplest possibility is that the population evolves to the phenotypically closest population to the one that would evolve if the assumption were true: that will often be an internal equilibrium with genetic variation. Maynard Smith (1981, 1982) made the general point about ESSs and population genetics, and we expect it to be true of inclusive fitness too. Uyenoyama and Feldman (1982) is just one example of population genetic models finding close results with internal equilibria. Sometimes, if the requisite average behavior cannot be achieved under the available genetic variation, there may be scope for intransitivity and for continual flux in gene frequencies. These rough guesses represent food for future theoretical thought and indicate how the equilibrium behavior under the probabilistic mixing assumption may turn out to be useful in understanding a system when that assumption is false.

We expect that the importance of probabilistic mixtures of phenotypes may extend to more general scenarios in which the genetic component of the variability in how individuals act on any given occasion is proportionally low (which implies the δ‐weak selection of Wild & Traulsen, 2007), because it removes the assortation effect of r. We expect this scenario to be the norm for populations near equilibria (or, more precisely, near a point at which a monomorphic population is uninvadeable by any one of set of mutations that code for all nearby phenotypes), where it is usually reasonable to suppose we study organisms (Birch, 2017a,b; Fisher, 1930; Grafen, 1985).

## 6 CONCLUSION

Empirical successes provide some assurance that the working hypothesis of inclusive fitness is by and large satisfactory. Here, we hope to have lent some formal support for such assurance. Further, we hope that our paper will present future modelers with a mathematical articulation of biologists’ intuitions about inclusive fitness under additivity and show how it can be extended on mild assumptions to provide useful guidance in more general situations.

## CONFLICT OF INTERESTS

The authors declare no competing interests.

## AUTHOR CONTRIBUTION

**Samuel R. Levin:** Conceptualization (equal); Formal analysis (equal); Methodology (equal); Writing–original draft (equal); Writing–review and editing (equal). **Alan Grafen:** Conceptualization (equal); Formal analysis (equal); Methodology (equal); Writing–original draft (equal); Writing–review and editing (equal).

## Data Availability

There are no data to be archived.

## Appendix 1

### Okasha and Martens

Okasha and Martens (2016b) analyze the simple cooperation game described in the text, in which an altruist donates *b* to its partner at a cost *c*, and when two cooperators are paired, they each receive an additional benefit, *d*. With initial strategy set $\left\{C,D\right\}$, there are four payoffs that we write formally as

(A1) $$\left(\begin{array}{cc} V\left(D,D\right) & V\left(D,C\right)\\ V\left(C,D\right) & V\left(C,C\right) \end{array}\right)=\left(\begin{array}{cc} 0 & b\\ ‐c & b‐c+d \end{array}\right).$$

We now invoke our probabilistic mixing assumption to allow strategies of the form $\phi \left(\pi \right)$ to represent playing D with probability 1‐π and C with probability π. Then, we extend the payoff function for mixes in terms of the original values as follows,

(A2) V(ϕ(σ),ϕ(π))=(1‐σ)(1‐π)V(D,D)+(1‐σ)πV(D,C)+σ(1‐π)V(C,D)+σπV(C,C).

To find an equilibrium strategy in the extended set, we seek a probability, 0<π<1 such that

$$V\left(\phi \left(\sigma \right),\phi \left(\pi \right)\right)\leq V\left(\phi \left(\pi \right),\phi \left(\pi \right)\right)\;\text{for all}\;\sigma \in \left[0,1\right].$$

Differentiating with respect to σ and solving for π when σ=π produces a well‐known result for an internal extremum (Grafen, 1979; Hines and Maynard Smith, 1979) in new notation that

(A3) $$\pi^{*}=‐\frac{rb‐c}{(1+r)d},$$

and note this is a maximum of payoffs if d<0 but a minimum if d>0. Thus, a mixed equilibrium relies on d<0, when there is a range of rb‐c values from 0 to $‐\left(1+r\right)d$ over which an internal mixture is stable. If rb‐c is negative, cooperation is absent from the equilibrium, while if it is above this range then all individuals cooperate. When d>0 and rb‐c is between $‐\left(1+r\right)d$ and 0, there are two local equilibria at the extremes, and again lower and higher values of rb‐c produce all Defect and all Cooperate, respectively.

This example has shown how payoff functions for scalar strategies need to be extended to probabilistic mixtures to implement the probabilistic mixing assumption. Inclusive fitness in this case is defined for a strategy $\phi \left(\sigma \right)$ in a population playing π by adding the payoff to s $\phi \left(\sigma \right)$ against a π incumbent, and adding r times the difference it makes to a π incumbent that the actor is playing σ not π, as follows,

(A4) $$U_{\mathrm{IF}}\left(\sigma ,\pi \right)=V\left(\phi \left(\sigma \right),\phi \left(\pi \right)\right)+r\left(V\left(\phi \left(\pi \right),\phi \left(\sigma \right)\right)‐V\left(\phi \left(\pi \right),\phi \left(\pi \right)\right)\right)=\left(\pi \left(b‐c\right)+\pi^{2}d\right)+\left(\sigma ‐\pi \right)\left(rb‐c+\pi \left(1+r\right)d\right),$$

where in the second line the first main bracket shows the payoff to $\phi \left(\pi \right)$ against itself, and the cofactor of $\left(\sigma ‐\pi \right)$ shows the inclusive fitness effect of one player deviating from π toward Cooperate against a population playing π. The rb‐c term is the familiar effect from additive models. Nonadditivity appears by regarding the effect of d as contributing d to self (with relatedness of 1) and d to the partner (with relatedness r), hence the factor 1+r. The factor π appears because this is the chance that the nonadditive gain will be made when the population plays π. Thus, the inclusive fitness makes complete sense. It has a turning point at an internal value of π only if the cofactor of σ‐π equals zero, which is a maximum only if d is negative, because then increasing σ above π results in a decrease in the player's fitness. The cofactor of σ‐π equaling zero immediately yields the solution for $\pi^{*}$ given above on dynamic grounds.

Thus, an inclusive fitness analysis, using our probabilistic mixing assumption and using the incumbent in place of Hamilton's “nonsocial” phenotype, yields a very satisfying analysis and interpretation of this two‐player game played between relatives. The probabilistic mixing assumption has the consequence that for inclusive fitness purposes we regard the partner as playing the incumbent strategy. Thus, this model supports the idea that we can consider organisms, at equilibrium, to appear as though maximizing their inclusive fitness (Okasha, 2018; Okasha & Martens, 2016b). The calculations effectively repeat those of Grafen (1979), but we articulate the arguments more fully.

## Appendix 2

### Lehmann et al.

Following Lehmann et al. (2015), and the assumptions outlined in our main text, we consider an infinite island model of haploid individuals on patches of size N. We extend Lehmann et al.’s (2015) analysis, and consider a mutant strategy $\tilde{y}$, in which a mutant displays the deviant behavior with some small probability, ε, and otherwise, with probability $\left(1‐\varepsilon \right)$, behaves like a resident (x): $\tilde{y}=\left(1‐\varepsilon \right)x+\varepsilon y$

#### First order conditions

We can rewrite Equation (10), by the definition of q and p from Lehmann et al. (2015), as,

(A5) $$W\left(\tilde{y},x\right)=\sum_{k=1}^{N}p_{k}\left(\tilde{y},x\right)w\left(\tilde{y},{\tilde{y}}^{\left(k‐1\right)}x^{\left(N‐k\right)},1_{x}\right),$$

where $p_{k}$ is the probability that a randomly drawn mutant has k‐1 other lineage members in its patch. A strategy x is uninvadable if, given x, $\tilde{y}=x$ is a local maximum of $W\left(\tilde{y},x\right)$.

In a slight abuse of notation, we will write partial derivatives of functions of $\tilde{y}$ with respect to y and suppress the functional dependence $\tilde{y}\left(y\right).$ When we come to unpack this expression for W, $p_{k}$, and w, which we do when we require x and y to be single real numbers for computational purposes, we will face and resolve the question of how these functions are extended when we allow their first argument to be a probabilistic combination of real numbers rather than a single real number. For the first example W, the definition of W for a general space from Lehmann et al. (their Equation 3) suffices for one stage of unpacking:

(A6) $$\frac{\partial W\left(\tilde{y},x\right)}{\partial y}{|}_{y=x}=\sum_{k=1}^{N}\frac{\partial }{\partial y}p_{k}\left(\tilde{y},x\right)w\left(\tilde{y},{\tilde{y}}^{\left(k‐1\right)}x^{\left(N‐k\right)},1_{x}\right){|}_{y=x}$$

$$+\sum_{k=1}^{N}p_{k}\left(\tilde{y},x\right)\frac{\partial }{\partial y}w\left(\tilde{y},{\tilde{y}}^{\left(k‐1\right)}x^{\left(N‐k\right)},1_{x}\right){|}_{y=x}.$$

The first term is equal to 0 because at y=x we can factor out the fitness term, and $\sum_{k=1}^{N}\frac{\partial }{\partial y}p_{k}\left(\tilde{y},x\right)=\partial \left(1\right)=0$

Turning to the second term, we unpack w by regarding it as a an average over the different realizations of $\tilde{y}$, and so now allow for a total k mutants and an independent chance, ε, of each of them displaying the deviant behavior. This gives:

(A7) $$\frac{\partial W\left(\tilde{y},x\right)}{\partial y}{|}_{y=x}=\sum_{k=1}^{N}p_{k}(\tilde{y},x)\frac{\partial }{\partial y}\sum_{h=0}^{k}\left(\begin{array}{c} k\\ h \end{array}\right)\varepsilon^{h}{\left(1‐\varepsilon \right)}^{k‐h}\left(\frac{h}{k}w\left(y,x^{\left(n‐h\right)}y^{\left(h‐1\right)},1_{x}\right)+\frac{k‐h}{k}w\left(x,x^{\left(n‐h‐1\right)}y^{\left(h\right)},1_{x}\right)\right){|}_{y=x}.$$

where h is the number of mutants displaying the deviant behavior. We can express the binomial as,

(A8) $$\left(\begin{array}{c} k\\ h \end{array}\right)\varepsilon^{h}{\left(1‐\varepsilon \right)}^{k‐h}\approx \left(\begin{array}{cc} \left(1‐k\varepsilon +O\left(\varepsilon^{2}\right)\right) & h=0\\ k\varepsilon +O(\varepsilon^{2}) & h=1\\ O(\varepsilon^{2}) & h\geq 2 \end{array}\right).$$

Eliminating higher order terms of ε gives:

(A9) $$\begin{array}{cc} \frac{\partial W\left(\tilde{y},x\right)}{\partial y}{|}_{y=x}\\ & =\sum_{k=1}^{N}p_{k}\left(\tilde{y},x\right)\frac{\partial }{\partial y}\left[\left(1‐k\varepsilon \right)w\left(x,x^{\left(N‐1\right)},1_{x}\right)+k\varepsilon \left(\frac{1}{k}w\left(y,x^{\left(N‐1\right)},1_{x}\right)+\frac{k‐1}{k}w\left(x,x^{\left(N‐2\right)}y,1_{x}\right)\right)\right]{|}_{y=x}+O\left(\varepsilon^{2}\right). \end{array}$$

We now adopt the notational convention of Lehmann et al. (2015, page 1862) that $w\left(y,x_{‐1},1_{x}\right)$ should be regarded as having N+1 arguments for the purpose of differentiation. Thus, we can take a partial derivative of up to the N+1th argument (though only actually use up to N). This allows us to take the derivative of one individuals offspring number with respect to the behavior of other single members of the group. By permutation invariance, and following Lehmann et al. (2015) in denoting $w_{j}$ as the derivative of w with respect to its *j*th argument, we get:

(A10) $$\frac{\partial W\left(\tilde{y},x\right)}{\partial y}{|}_{y=x}=\varepsilon \left(\sum_{k=1}^{N}p_{k}\left(\tilde{y},x\right)\left(k‐1\right)w_{N}\left(x,x^{\left(N‐1\right)}y,1_{x}\right)\right)+\varepsilon \left(\sum_{k=1}^{N}p_{k}\left(\tilde{y},x\right)w_{1}\left(y,x^{\left(N‐2\right)},1_{x}\right)\right){|}_{y=x}.$$

And from the definition of relatedness following Lehmann et al. (2015), $r\left(\tilde{y},x\right)=\sum_{k=1}^{N}\frac{p_{k}\left(\tilde{y},x\right)\left(k‐1\right)}{\left(N‐1\right)}$ we obtain a first order condition of:

(A11) $$\begin{array}{cc} \frac{\partial W\left(\tilde{y},x\right)}{\partial y}{|}_{y=x} & =\varepsilon \left(w_{1}\left(x,x^{\left(N‐1\right)},1_{x}\right)+\left(N‐1\right)\sum_{k=1}^{N}\frac{p_{k}\left(x,x\right)\left(k‐1\right)}{\left(N‐1\right)}w_{N}\left(x,x^{\left(N‐1\right)},1_{x}\right)\right)\\ & =\varepsilon \left[w_{1}\left(x,x^{\left(N‐1\right)},1_{x}\right)+\left(N‐1\right)r\left(x,x\right)w_{N}\left(x,x^{\left(N‐1\right)},1_{x}\right)\right]=0. \end{array}$$

#### Second order condition

The second order condition is given by the second derivative:

(A12) $$\begin{array}{cc} \frac{\partial^{2}W\left(\tilde{y},x\right)}{\partial y^{2}}{|}_{y=x} & =\sum_{k=1}^{N}\frac{\partial^{2}}{\partial y^{2}}p_{k}\left(\tilde{y},x\right)w\left(\tilde{y},y^{\left(k‐1\right)}x^{\left(N‐k\right)},1_{x}\right){|}_{y=x}\\ & +2\sum_{k=1}^{N}\frac{\partial }{\partial y}p_{k}\left(\tilde{y},x\right)\frac{\partial }{\partial y}w\left(\tilde{y},y^{\left(k‐1\right)}x^{\left(N‐k\right)},1_{x}\right){|}_{y=x}\\ & +\sum_{k=1}^{N}p_{k}\left(\tilde{y},x\right)\frac{\partial^{2}}{\partial y^{2}}w\left(\tilde{y},y^{\left(k‐1\right)}x^{\left(N‐k\right)},1_{x}\right){|}_{y=x}. \end{array}$$

The first term of the RHS of the equation equals 0 because at y=x we can factor out the fitness term, and $\sum_{k=1}^{N}\frac{\partial^{2}}{\partial y^{2}}p_{k}\left(\tilde{y},x\right)=\partial^{2}\left(1\right)=0$

Turning to the second term, we already have the partial derivative of w (above) as: $\varepsilon kw_{j}\left(y,x^{\left(N‐1\right)},1_{x}\right)$. We unpack by using the definition of $p_{k}$ from Lehmann et al. (Lehmann et al. (2015, box 2) and write:

(A13) $$\frac{\partial }{\partial y}p_{k}\left(\tilde{y},x\right)=\frac{\partial }{\partial y}\frac{kt_{k}\left(\tilde{y},x\right)}{\sum_{h=1}^{N}ht_{h}\left(\tilde{y},x\right)}=\frac{\frac{\partial }{\partial y}kt_{k}\left(\tilde{y},x\right)\left(\sum_{h=1}^{N}ht_{h}\left(\tilde{y},x\right)\right)‐kt_{k}\left(\tilde{y},x\right)\frac{\partial }{\partial y}\sum_{h=1}^{N}ht_{h}\left(\tilde{y},x\right)}{{\left(\sum_{h=1}^{N}ht_{h}\left(\tilde{y},x\right)\right)}^{2}},$$

where $t_{k}\left(\tilde{y},x\right)$ is the number of demographic periods (“sojourn time”) for which the lineage consists of k individuals. To get an expression for $\frac{\partial }{\partial y}t_{k}\left(\tilde{y},x\right)$, we use the matrix, Q, from which $t_{k}$ is derived, as defined in the Supplementary Material of Lehmann et al. (2015, equation A1)). Q is a matrix whose i, jth entry is the probability a patch with j mutants becomes a patch with i mutants in the next demographic period. To obtain the formula, we need a symbol $R_{i‐f,j‐h,h}\left(y,x\right)$ for the probability that the j‐h nondeviant mutants contribute i‐f individuals to the next time step. The probability of going from j to i mutants is then

(A14) $$\begin{array}{cc} Q_{\mathrm{ij}}\left(\tilde{y},x\right) & =\pi_{j0}Q_{\mathrm{ij}}\left(x,x\right)+\sum_{h=1}^{j‐1}\pi_{\mathrm{jh}}\left(1‐\sum_{k=1}^{N}Q_{k,h}\left(y,x\right)+R_{i\left(j‐h\right)h}\left(y,x\right)\right)\\ & +\sum_{h=1}^{j‐1}\pi_{\mathrm{jh}}\sum_{f=1}^{i‐1}\left(Q_{f,h}\left(y,x\right)+R_{\left(i‐f\right)\left(j‐h\right)h}\left(y,x\right)\right)\\ & +\sum_{h=1}^{j‐1}\pi_{\mathrm{jh}}\left(Q_{i,h}\left(y,x\right)+1‐\sum_{k=1}^{N}R_{k\left(j‐h\right)h}\left(y,x\right)\right)+\pi_{\mathrm{jj}}Q_{\mathrm{ij}}\left(y,x\right), \end{array}$$

where h represents the number out of a total j mutants that display the behavior y, and $\pi_{\mathrm{jh}}$ is the probability that a group with j mutants will have h individuals displaying the behavior. The Q matrices capture individuals contributed by the deviant displaying mutants, and the R matrices capture mutant individuals contributed by mutants acting as residents.

Now, we apply our assumptions that only a small fraction ε of mutants display and that the chances of displaying are all independent. This gives us

(A15) $$\pi_{\mathrm{jh}}=\left(\begin{array}{c} i\\ h \end{array}\right)\varepsilon^{h}{\left(1‐\varepsilon \right)}^{j‐h}\approx \left(\begin{array}{cc} \left(1‐j\varepsilon +O(\varepsilon^{2})\right) & h=0\\ j\varepsilon +O(\varepsilon^{2}) & h=1\\ O(\varepsilon^{2}) & h\geq 2 \end{array}\right).$$

Substituting and eliminating higher orders of ε, we find:

(A16) $$Q_{\mathrm{ij}}\left(\tilde{y},x\right)=Q_{\mathrm{ij}}\left(x,x\right)+j\varepsilon \left(‐Q_{\mathrm{ij}}\left(x,x\right)+1‐\sum_{f=i+1}^{N}Q_{f,1}\left(y,x\right)+1‐\sum_{k=i+1}^{N}R_{k\left(j‐1\right)1}\left(y,x\right)\right).$$

Let A, B and C be N×N matrices of the form

$$\left[\begin{array}{ccccc} 0 & 1 & 1 & \ldots & 1\\ 0 & 0 & 1 & \ldots & 1\\ \vdots & \vdots & \vdots & \vdots & \vdots \\ 0 & 0 & 0 & \ldots & 1\\ 0 & 0 & 0 & \ldots & 0 \end{array}\right],\left[\begin{array}{ccccc} 1 & 1 & 1 & \ldots & 1\\ 0 & 0 & 0 & \ldots & 0\\ \vdots & \vdots & \vdots & \vdots & \vdots \\ 0 & 0 & 0 & \ldots & 0 \end{array}\right],\text{and}\left[\begin{array}{ccccc} 0 & 1 & 0 & \ldots & 0\\ 0 & 0 & 1 & \ldots & 0\\ \vdots & \vdots & \vdots & \vdots & \vdots \\ 0 & 0 & 0 & \ldots & 1\\ 0 & 0 & 0 & \ldots & 0 \end{array}\right],$$

respectively. Let **J** be the *N* × *N* matrix with the vector *j* as the diagonal and otherwise zeroes, *l* be the *N* × *N* matrix of ones, and $R_{1}$ be the matrix with entries $R_{k,j,1}$ (representing the probability a patch with *j* nondeviant mutants, in the presence of *l* deviant mutant, becomes a patch with k mutants). Then, we can write *Q* as:

(A17) $$Q\left(\tilde{y},x\right)=Q\left(x,x\right)+\varepsilon \left(‐Q\left(x,x\right)+1‐A\left(Q\left(y,x\right)B\right)+1‐\left(AR_{1}\left(y,x\right)\right)C\right)J.$$

Now, $t_{k}\left(\tilde{y},x\right)$ is taken from the first column of ${\left(I‐Q\left(\tilde{y},x\right)\right)}^{‐1}$. From the binomial theorem for matrices (Arias et al., 1990),

(A18) $${\left(I‐Q\left(\tilde{y},x\right)\right)}^{‐1}={\left(I‐Q\left(x,x\right)\right)}^{‐1}‐\varepsilon {\left(I‐Q\left(x,x\right)\right)}^{‐1}\left(\left(Q\left(x,x\right)‐\Omega \right)J\right){\left(I‐Q\left(x,x\right)\right)}^{‐1}+o\left(\varepsilon^{2}\right),$$

where $\Omega =1+A\left(Q\left(y,x\right)B\right)‐1+\left(AR_{1}\left(y,x\right)\right)C$. Thus, writing $T\left(\tilde{y},x\right)$ as the matrix $\left(I‐Q\left(\tilde{y},x\right)\right)$ that contains as its first column the vector of $t_{k}$'s, we write:

(A19) $$\frac{\partial }{\partial y}T\left(\tilde{y},x\right)=‐\varepsilon \left(T\left(x,x\right)\frac{\partial }{\partial y}\left(\left(A\left(Q\left(y,x\right)B\right)+\left(AR_{1}\left(y,x\right)\right)C\right)J\right)\left(T\left(x,x\right)\right)\right).$$

It follows from our assumption that x and y are real numbers that the derivatives above are bounded except at a countable number of points, and thus, the above expression is of order ε. Since the $t_{k}$'s are taken from Equation A20, the derivative of $t_{k}$ with respect to y is of order ε, which means that Equation A14 is of order ε, and thus line 3 of equal A13 is of order $\varepsilon^{2}$. This gives:

(A20) $$\frac{\partial^{2}W\left(\tilde{y},x\right)}{\partial y^{2}}{|}_{y=x}=\sum_{k=1}^{N}p_{k}\left(\tilde{y},x\right)\frac{\partial^{2}}{\partial y^{2}}w\left(\tilde{y},y^{\left(k‐1\right)}x^{\left(N‐k\right)},1_{x}\right){|}_{y=x}.$$

Following the same steps as above (equations (A7), (A8), (A9), (A10), (A11)), we can write the second order condition as:

(A21) $$\begin{array}{cc} \frac{\partial^{2}W\left(\tilde{y},x\right)}{\partial y^{2}}{|}_{y=x} & =\sum_{k=1}^{N}p_{k}\left(\tilde{y},x\right)\frac{\partial^{2}}{\partial y^{2}}\left[\left(1‐k\varepsilon \right)w\left(x,x^{\left(N‐1\right)},1_{x}\right)+k\varepsilon \left(\frac{1}{k}w\left(y,x^{\left(N‐1\right)},1_{x}\right)+\frac{k‐1}{k}w\left(x,x^{\left(N‐2\right)}y,1_{x}\right)\right)\right]{|}_{y=x}\\ & =\varepsilon \left[w_{11}\left(x,x^{\left(N‐1\right)},1_{x}\right)+\left(N‐1\right)r\left(x,x\right)w_{\mathrm{NN}}\left(x,x^{\left(N‐1\right)},1_{x}\right)\right]<0. \end{array}$$

#### Expected utility maximization

Turning to our expected utility function, $u_{\mathrm{IF}}$ defined in equation (8), we can rewrite it with the focal strategy of $\tilde{y}$ as

(A22) $$u_{\mathrm{IF}}\left(\tilde{y},x\right)=w\left(\tilde{y},x^{\left(N‐1\right)}1_{,}x\right)+\left(N‐1\right)r\left(\tilde{y},x\right)\left[w\left(x,x^{\left(N‐2\right)}\tilde{y},1_{x}\right)‐w\left(x,x^{\left(N‐2\right)}x,1_{x}\right)\right]$$

and, as equations ((A13), (A14), (A15), (A16), (A17), (A18), (A19)) show that we can replace $r\left(\tilde{y},x\right)$ with $r\left(x,x\right)$, as part of eliminating terms of higher order of ε, this leads to first order condition at y=x of

$$w_{1}\left(x,x^{\left(N‐1\right)},1_{x}\right)+\left(N‐1\right)r\left(x,x\right)w_{N}\left(x,x^{\left(N‐1\right)},1_{x}\right)=0,$$

and second order condition of

$$w_{11}\left(x,x^{\left(N‐1\right)},1_{x}\right)+\left(N‐1\right)r\left(x,x\right)w_{\mathrm{NN}}\left(x,x^{\left(N‐1\right)},1_{x}\right)<0,$$

which match the uninvadability conditions in Equations (A12) and (A22).

Thus, the first and second order conditions for uninvadability and utility maximization are identical when our inclusive fitness is used as utility.
